# Protective Effects of *Salvia miltiorrhizae* on Multiple Organs of Rats with Obstructive Jaundice

**DOI:** 10.1155/2009/602935

**Published:** 2009-08-06

**Authors:** Zhang Ling, Zhang Xiping, Qiu Fengmei, Yan Ping, Cheng Qihui

**Affiliations:** ^1^The Cardiovascular Research Laboratory, Lakehead University, 290 Munro Street, Thunder Bay, ON, Canada P7A 7 T1; ^2^Department of General Surgery, Hangzhou First People's Hospital, Hangzhou 310006, Zhejiang, China; ^3^Zhejiang University of Traditional Chinese Medicine, Hangzhou 310053, Zhejiang, China; ^4^Department of Gynaecology and Obstetrics, Hangzhou First People's Hospital, Hangzhou 310006, Zhejiang, China

## Abstract

*Purpose*. we aim to explore the protective effects of *Salvia miltiorrhizae* injection on multiple organs of obstructive jaundice (OJ) rats through observing the impact of this injection on the pathological alterations in these organs and the contents of endotoxin, PLA_2_, and TNF-*α* in the blood. *Methods*. A total of 90 mice were randomly divided into sham-operated group, model-control group, and *Salvia miltiorrhizae*-treated group (*n* = 30). According to the duration of postoperative administration, each group was further divided into two subgroups, namely, 21 d subgroup (consecutive administration for 21 d, *n* = 15) and 28 d subgroup (consecutive administration for 28 d, *n* = 15). After administration, the pathological alterations in multiple organs were observed and the contents of endotoxin, PLA_2_, and TNF-*α* in the blood were determined. *Results*. Compared to model control group, the number of dead rats in treated group decreased though there was no statistical difference between the two groups. The pathological alterations in the liver, kidney, and spleen in treated group showed varying degrees of mitigation. At all time points, the contents of plasma endotoxin declined significantly. On day 28, plasma PLA_2_ content in treated group was significantly lower than that in model-control group. *Conclusion*. *Salvia miltiorrhizae* injection is able to obviously reduce the contents of inflammatory mediators in the blood of OJ rats and exert some protective effects on multiple organs of these rats.

## 1. Introduction

It is currently believed that obstructive jaundice (OJ) can induce functional damage to the liver, spleen, lymphocytes, and gastrointestinal mucosa as well as cardiac hemodynamic dysfunction [1–4]. However, the mechanisms underlying OJ-induced multiple organ damage are still unclear, and there are many controversial hypotheses to explain this phenomenon. It is generally believed that endotoxin, inflammatory mediators, and increased bile acids contribute to OJ-induced liver or other organs injuries. Moreover, elevated intrabiliary duct pressure and portal vein pressure are also important factors contributing to this injury.

*S. miltiorrhizae* is a kind of traditional Chinese drug commonly used for activating blood circulation, the research on the effect of it in the treatment of OJ has been reported [5, 6]. *S. miltiorrhizae* injection is the extraction of wild Salvia roots, the main active ingredients include danshensu, salvianolic acid as well as tanshinone, dihydrotanshinone, and cryptotanshinone, which are able to protect endothelial cells, fight against inflammation, and prevent lipid peroxidation and calcium overload [7, 8]. Some studies have shown that when *S. miltiorrhizae* is used to treat liver and kidney damage in OJ rats, it can exert some therapeutic effects against this damage through protecting hepatic cells, maintaining liver function, as well as reducing the damage to renal cortex and renal function [4, 6, 9, 10]. Moreover, *S. miltiorrhizae* also shows some therapeutic effects on OJ patients [11]. Although *S. miltiorrhizae* has been used to treat single organ injury complicating OJ in few studies [6, 11], a comprehensive study on the treatment of OJ-induced multiorgan damage with *S. miltiorrhizae* is lacking. For this reason, the purpose of this study is to make up for this deficiency. Through observing the impact of *S. miltiorrhizae* injection on the pathological alterations in multiple organs and the contents of inflammatory mediators in the blood of OJ rats, we explored the protective effects of *S. miltiorrhizae* on these organs.

## 2. Materials and Methods

### 2.1. Materials

A total 90 healthy male SD rats of clean grade, weighing between 270 and 330 g, were provided by the Laboratory Animal Research Center of the Zhejiang University of Traditional Chinese Medicine (China); Sodium taurocholate and sodium pentobarbital were purchased from Sigma Corporation, USA. *S. miltiorrhizae* injection (each 10 mL vial contains active components equivalent to 15 g of the original medicine) was purchased from Chiatai Qingchunbao Pharmaceutical Co., Ltd. (China). Endotoxin ELISA Kit was purchased from Associates of Cape Cord (USA), the calculation unit for content is EU/mL. Serum TNF-*α* ELISA Kit was purchased from Shanghai Senxiong Technology Industry Co., Ltd. (China), the calculation unit for content is ng/L (pg/mL). Serum secretory phospholipase A_2_ enzyme Assay ELA kit (PLA_2_) was purchased from R&D system Ins (USA), the calculation unit for content is U/mL.

### 2.2. Methods

#### 2.2.1. Animal Grouping

90 rats were utilized for OJ-associated experiments and randomly divided into sham-operated, model-control, and treated group (*n* = 30), which were further randomly subdivided into 21 d and 28 d groups (*n* = 15) according to time duration after operation.

#### 2.2.2. Preparation of OJ Models and Associated Therapeutic Regimen

After rats were anesthetized with an intraperitoneal injection of 2.5% sodium pentobarbital (0.2 mL/100 g), the abdominal cavity was opened to identify and dissociate common bile duct along the hepatoduodenal ligament. For rats in the model-control groups and the treated groups, the proximal end of common bile duct was double-ligated with surgical threads, common bile duct was cut off, and a layered suture of the abdominal wall was performed to close the abdominal cavity. For rats in the sham-operated groups, common bile duct was only dissociated but not ligated, and a layered suture of the abdominal wall was also performed to close the abdominal cavity. An intraperitoneal injection of *S. miltiorrhizae* injection at a dose of 0.2 mL/100 g/d [12–15] was given to rats in the treated groups while equal volume of physiological saline solution was used in the sham-operateds and the model-control groups. Injection was maintained until the end of the 21st day and 28th day observation period in the corresponding groups.

#### 2.2.3. Specimen Collection

On the 21st day and 28th day after operation, all alive rats were anesthetized with 2.5% sodium pentobarbital and killed mercyfully. Blood samples and tissue specimens of liver, lung, spleen, kidney, thymus, intestinal muscoa, and mesenteric lymph node were then collected, respectively.

#### 2.2.4. Determination of Experimental Parameters

**(1) Observation of Mortality Rate and Pathological Changes**

The mortality rates of rats in various groups were recorded. The gross pathological changes and pathological changes under light microscopy of different tissues were observed, respectively.

**(2) Determination of the Levels of Plasma Endotoxin, PLA_2_, and Serum TNF-*α***

The determination of these parameters was conducted according to the instructions provided with the kits.

#### 2.2.5. Statistical Analysis

The compiled data were first put into Excel sheet, and then read SPSS15.0 for further analysis. Normal data were expressed as means (standard deviation) while abnormal data were expressed as medians (interquartile range). Analysis of variance and pairwise comparisons were used for normal data, whereas abnormal data were subjected to non-parametric test, among which Kruskal-Wallis H test was used for pairwise comparisons and Mann-Whitney U test for multiple comparisons. Yates' chi-square test (*χ* ^2^) was used for intergroup comparisons of mortality rates.

## 3. Results

### 3.1. Comparison of Mortality Rate

4 and 7 rats died in the model-control groups on 21st and 28th day, respectively; and 2 and 4 rats died in the treated groups on 21st and 28th day. On the 21st day, the mortality rates in the sham-operated groups were significantly lower than those in the model-control groups (*P* = .032); On 28 d, the mortality rate in the sham-operated group was significantly lower than those in both the model-control group (*P* = .006) and the treated group (*P* = .032), and the difference was statistically significant. Compared with model-control group, though the number of dead OJ rats in the treated group declined, no statistical difference was noted (*P* > .05).

### 3.2. Pathological Changes of the Multiple Organs Under Gross and Light Microscopy

#### 3.2.1. Liver

**(1) Sham-Operated Group**

**Gross**

The liver was normal and showed no obvious pathological changes.

**Under the Light Microscope**

**Figure 1:**
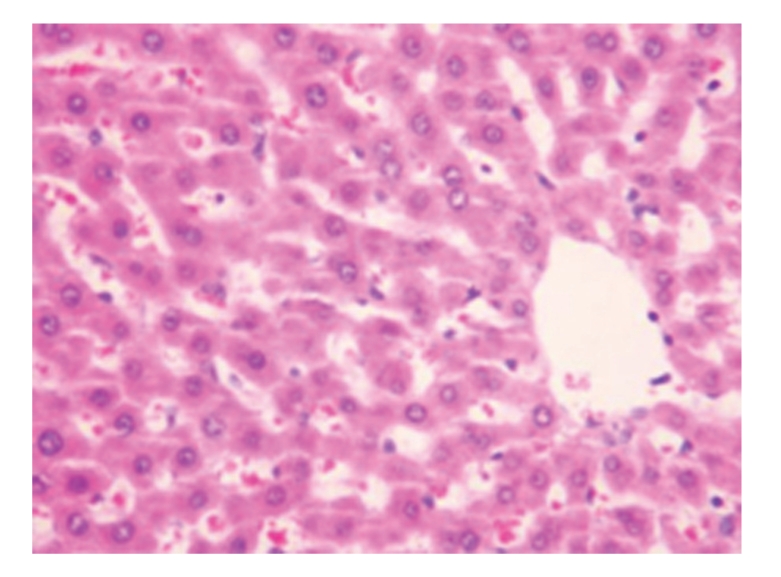
Sham-operated group 28 days; normal liver cells; HE × 200.

There was no marked difference among various time points after operation, and the liver was basically normal. The focal infiltrations of inflammatory cells were occasionally seen in few hepatic tissues, see Figure 1.

**(2) Model-Control Group**

**Gross**

The gross pathological changes manifested as an increase in size and hypertrophy. On 21st and 28th d, 80 percent of livers had a thickness of 0.8 cm. The section plane showed yellow plaques and was slightly oily. The texture of the liver became fragile, the bile ducts were dilated and enlarged, and even formed cysts (an average size of approximately 2 × 2 × 1 cm^3^).

**Under the Light Microscope**

**Figure 2:**
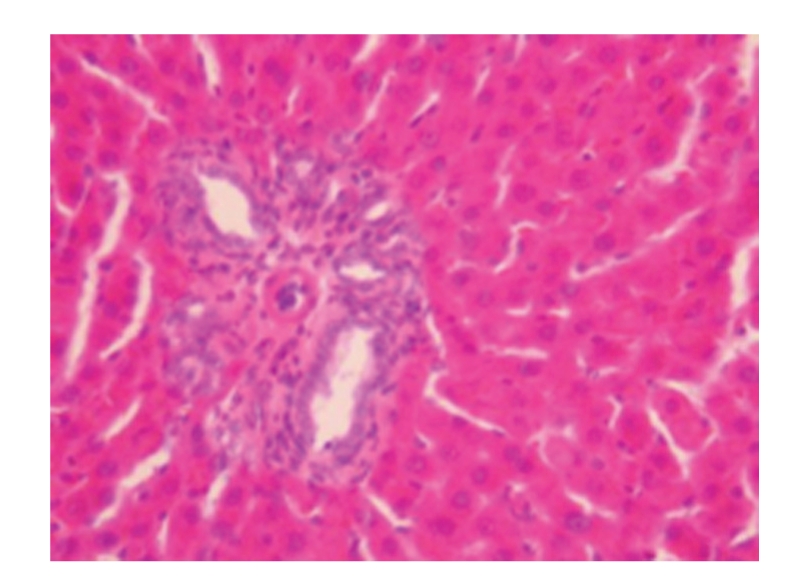
Model-control group 21 days; degeneration and swelling of hepatic cells as well as hyperplasia of bile ducts; HE × 200.

**Figure 3:**
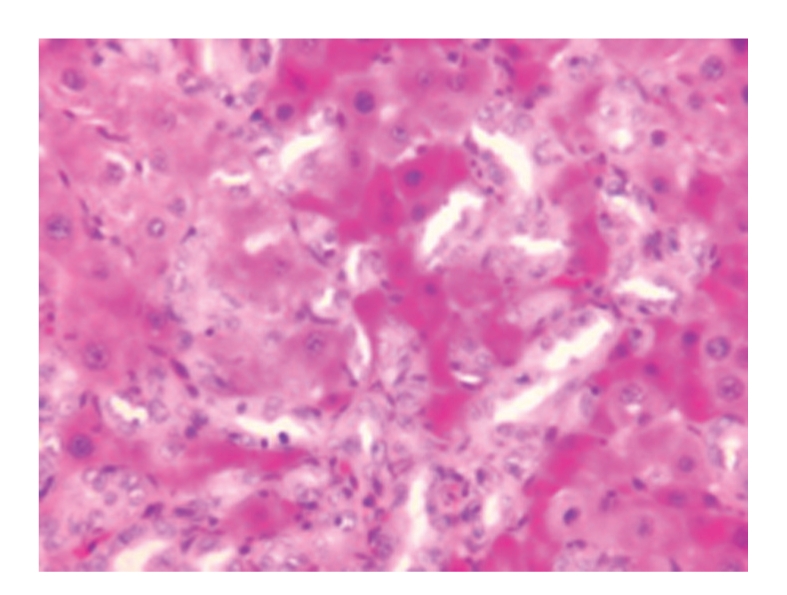
Model-control group 28 days; hyperplasia of small hepatic bile ducts and obvious degeneration of hepatic cells; HE × 200.

There were aggravated with the increase of postoperative duration. These pathological changes mainly manifested as swelling and obscure boundary in the majority of hepatic cells, the narrowing or partial disappearance of some hepatic sinusoids, as well as vascuolation and dissolution of hepatic cells in occasional part of the liver. The number of apoptotic bodies increased. The connective tissue in the most part of the portal area showed stellated hyperplasia. The bile ducts, whose surroundings revealed acute and chronic inflammatory cell infiltration, were enlarged and hyperproliferated. The limiting plate of hepatocytes were destroyed and showed focal necrosis, see Figures 2  and 3.

**(3) Treated Group**

**Gross**

In rats treated with *S. miltiorrhizae*, the liver was mildly enlarged in size, showed yellowish-brown color, and had a sharper edge than that in model-control group. The left and right hepatic ducts as well as the proximal common bile duct were dilated to form cysts that had a size smaller than those in model-control group.

**Under the Light Microscope**

The pathological changes showed no obvious difference between 21 and 28 days after operation, but there were mitigated to varying degrees when compared with those in the model-control group.

#### 3.2.2. Kidney

**(1) Sham-Operated Group**

**Gross**

No obvious abnormality was seen.

**Under the Light Microscope**

No obvious changes were seen in kidney, and an edema of renal tubular epithelial cells could be seen occasionally.

**(2) Model-Control Group**

**Gross**

Diffuse yellow staining of renal capsule, cortex, and medulla was seen. The renal cortex in nearly half of rats showed brownish-black color. The kidney showed no changes in size and texture.

**Under the Light Microscope**

**Figure 4:**
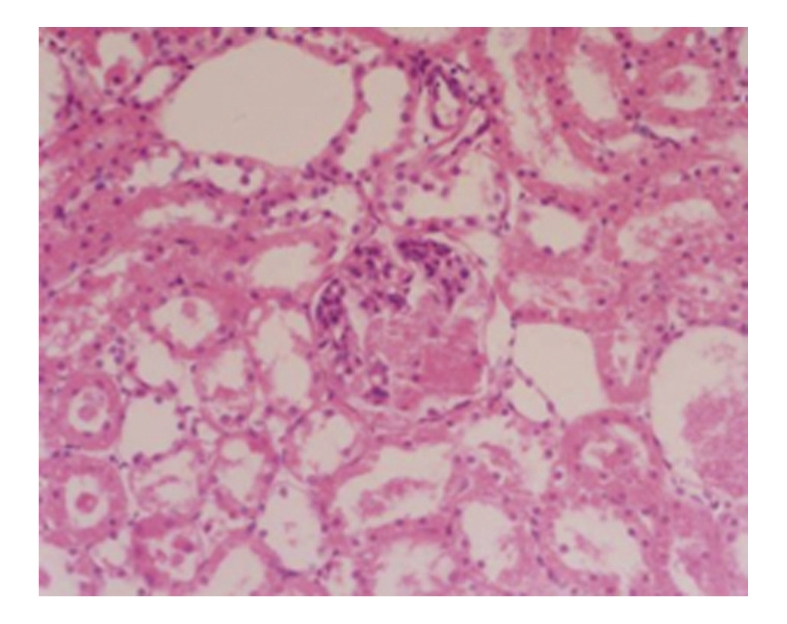
Sham-operated group 28 days; granular substances were seen in the space between glomerular capillaries as well as in the glomerulus and the cytoplasm of renal tubular epithelial cells, and patchy necrosis of renal tubular epithelium was also observed; HE × 200.

Swelling and necrosis of proximal or distal tubules, bile pigment deposition, bile pigment casts within the lumen of renal tubules, patchy necrosis and bile pigment deposition in renal tubules, and inflammatory cell infiltration as well as hyperplasia and edema of connective tissue in renal interstitium were seen. These pathological changes were aggravated with the prolongation of time, see Figure 4.

**(3) Treated Group**

**Gross**

Renal cortex in 80% of rats in treated group showed dark brown color, which was lighter than that in model-control group; renal medulla showed yellow color. The kidney showed no changes in size and texture.

**Under the Light Microscope**

**Figure 5:**
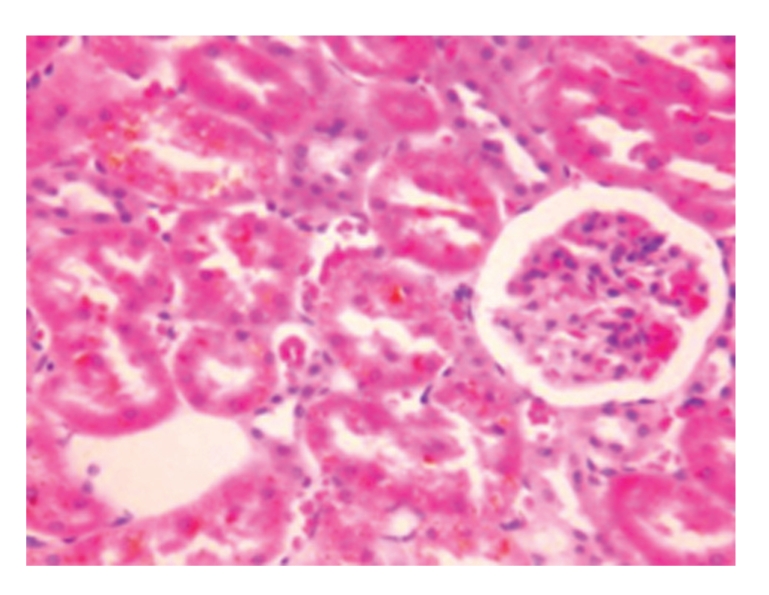
Treated group 21 days; obvious glomerular congestion and renal tubular degeneration; HE × 200.

**Figure 6:**
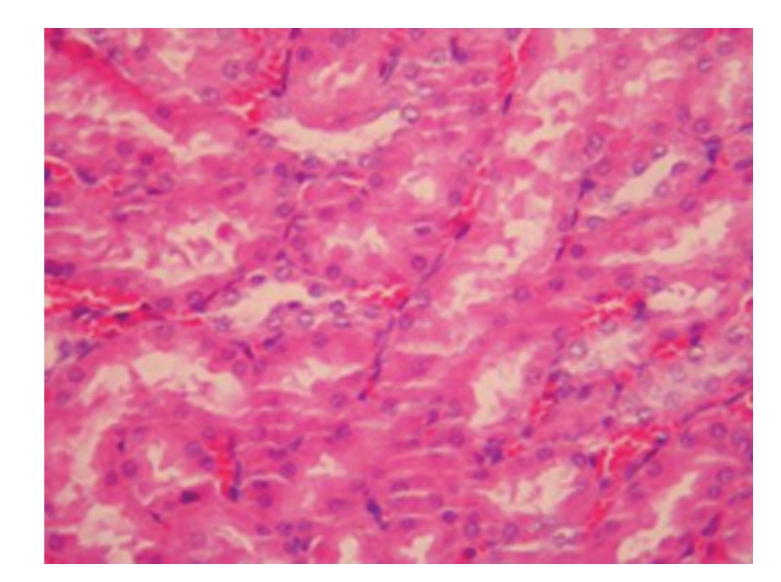
Treated group 28 days; turbidity and degeneration of renal tubular epithelial cells as well as vascular congestion; HE × 200.

Mainly manifested as hyperplasia of glomerular capillary endothelial cells, epithelial cells, mesangial cells, and basement membrane were milder than those in model-control group, see Figures 5  and 6.

#### 3.2.3. Lung

**(1) Sham-Operated Group**

**Gross**

The color and shape of the lungs were normal, and there was no exudate in the thoracic cavity.

**Under the Light Microscope**

There was no obvious difference in pathological changes. The majority of lung tissues showed normal structure. Edema and infiltrated inflammatory cells were seen in a very small part of pulmonary interstitium and alveolar space. Occasional widening of alveolar septum, capillary distention, and congestion were observed.

**(2) Model-Control Group**

**Gross**

Dark red plaques were seen in the lungs of some rats.

**Under the Light Microscope**

**Figure 7:**
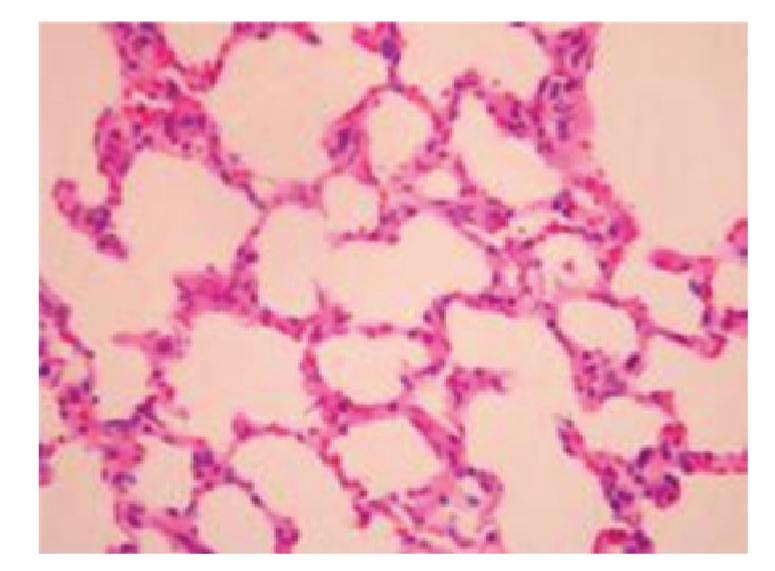
Sham-operated group 28 days; normal lung tissue; HE × 200.

The pathological alterations in model-control group were slightly aggravated with an increase in postoperative duration. On day 21, the lung of the majority of rats showed edema in the interstitium and alveolar wall as well as capillary distension in the alveolar wall while the lung of some rats showed widening of the alveolar septum. On day 28 in model-control group, the lung of the majority of rats showed hyperplasia and widening of interstitial fiber in the alveolar wall as well as edema and inflammatory cell infiltration in the interstitium and alveolar wall, see Figure 7.

**(3) Treated Group**

**Gross**

No marked difference in pathological changes was observed compared to those in model-control group.

**Under the Light Microscope**

**Figure 8:**
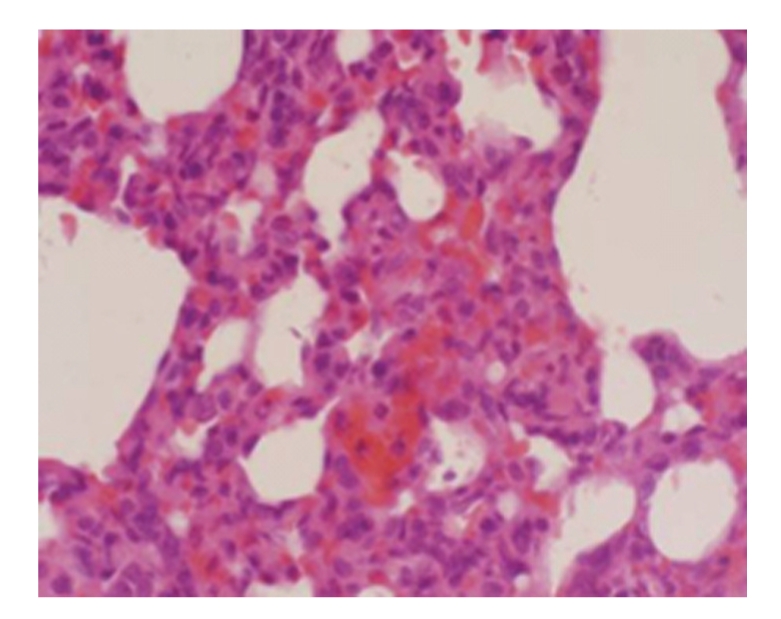
Treated group 28 days; capillary distension and congestion in the alveolar wall as well as inflammatory cell infiltration in the alveolar septum; HE × 400.

On day 21, the lung of the majority of rats showed edema in the alveolar wall and inflammatory cell infiltration in the interstitium. The lung of some rats showed hyperplasia and widening of the alveolar wall as well as vascular distention. The lung of individual rats was normal. On day 28, the lung of the majority of rats showed edema and inflammatory cell infiltration in the interstitium and alveolar wall as well as hyperplasia and widening of interstitial fiber while the lung of individual rats was normal. Overall, the pathological alterations in treated group were milder than those in model-control group, see Figure 8.

#### 3.2.4. Intestinal Mucosa

**(1) Sham-Operated Group**

**Gross**

No obvious abnormality was seen.

**Pathological Changes under Light Microscopy**

No obvious difference in pathological changes were observed. The intestinal mucosa was normal in the majority of rats. The intestinal mucosa epithelium was not intact in very few rats. Inflammatory cell infiltration was seen in proper layer.

**(2) Model-Control Group**

**Gross**

Yellow staining of the intestinal wall and peritoneum were seen in all rats.

**Pathological Changes under Light Microscopy**

No obvious difference in pathological changes was observed. On day 21 after operation, intestinal mucosa was not intact in the majority of rats, the edema of proper layer, submucous layer, and serosal layer was seen in the majority of rats, and very few rats showed no abnormality of the intestinal mucosa. On 28 d after operation, focal necrosis in intestinal mucosa epithelium, as well as the edema of proper layer, submucous layer, and serosal layer were seen in the majority of rats.

**(3) Treated Group**

**Gross**

No obvious difference was observed when compared to those in model-control group.

**Pathological Changes under Light Microscopy**

No obvious difference in pathological changes was observed among each time points after operation. Inflammatory cell infiltration was seen in proper layer, submucous layer, and serosal layer in the majority of rats. Intestinal mucosa was normal in some rats, and intestinal mucosa was not intact in very few rats. On 21 d after operation, some rats showed no abnormality of the intestinal mucosa; inflammatory cell infiltration was seen in proper layer in some rats; intestinal mucosa was not intact in very few rats. On 28 d after operation, some rats showed no abnormality of the intestinal mucosa; inflammatory cell infiltration was seen in proper layer, submucous layer, and serosal layer in some rats; and intestinal mucosa was not intact in very few rats.

#### 3.2.5. Spleen

**(1) Sham-Operated Group**

**Gross**

The morphology of spleen was normal, and no obvious pathological changes were seen.

**Pathological Changes under Light Microscopy**

The spleen was roughly normal in all rats.

**(2) Model-Control Group**

**Gross**

The spleen became enormous, with an average size of 4 × 1 × 1 cm. The texture of spleen became fragile, and the color of spleen became purple black.

**Pathological Changes under Light Microscopy**

**Figure 9:**
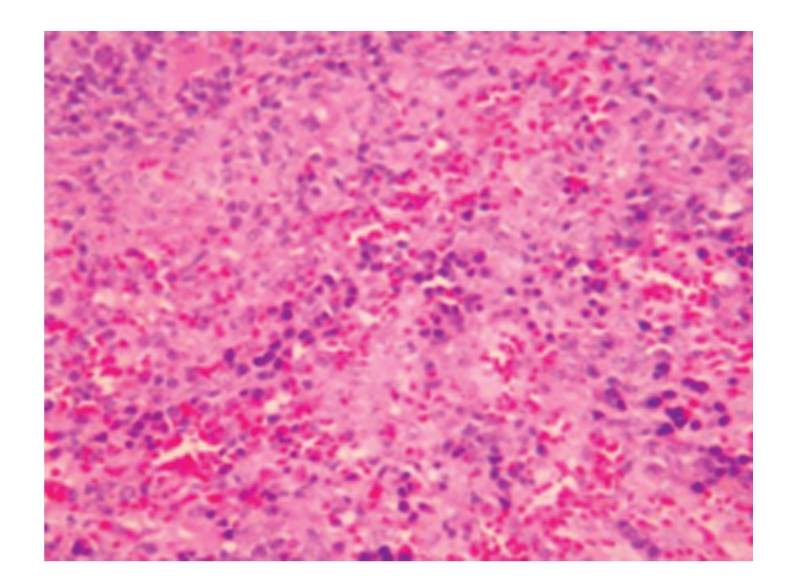
Sham-operated group 28 days; hyperplasia of cells in the splenic sinus, enlargement of blood sinus, inflammatory cell infiltration, and hemorrhage; HE × 200.

**Figure 10:**
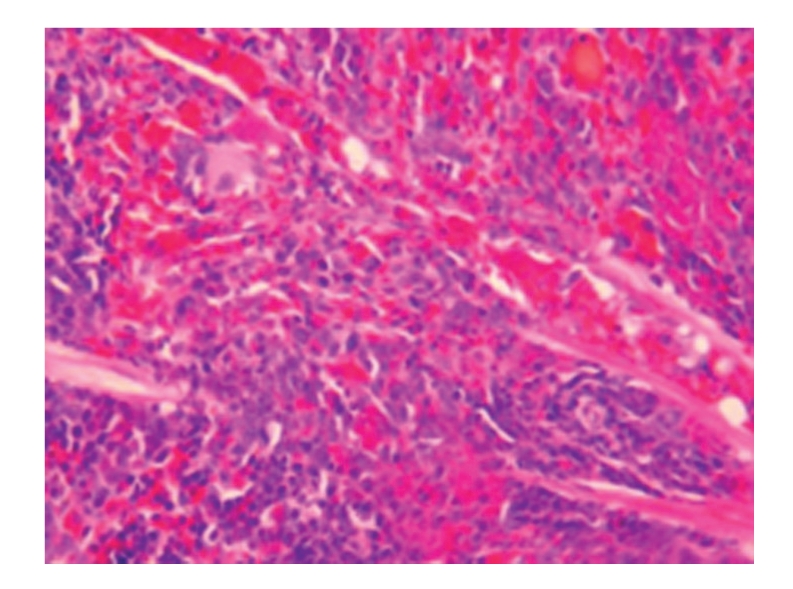
Sham-operated group 28 days; enlargement of splenic sinusoid and hyperplasia of fibrous tissue; HE × 200.

The fusion, enlargement, or spotty necrosis of follicular germinal centers in the white pulp of spleen, the hyperplasia of the fibrous tissue in the sinus, as well as splenic arteriolar sclerosis were seen in few rats. The spleen was roughly normal in some rats, see Figures 9  and 10.

**(3) Treated Group**

**Gross**

The size of spleen increased, with an average size of 3 × 1 × 0.5 cm.

**Pathological Changes under Light Microscopy**

**Figure 11:**
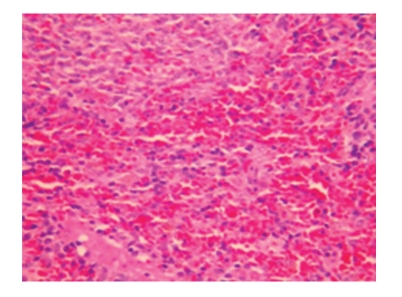
Treated group 28 days; enlargement and congestion of splenic sinusoid; HE × 200.

No significant difference in pathological changes was noted among each time point after operation. The spleen was roughly normal in the majority of rats. Splenic arteriolar sclerosis was seen in few rats, see Figure 11.

#### 3.2.6. Thymus

**(1) Sham-Operated Group**

**Gross**

The morphology of thymus was normal, and no obvious pathological changes were seen.

**Pathological Changes under Light Microscopy**

No significant difference in pathological changes was noted among each time point after operation. The thymic tissue was roughly normal in all rats.

**(2) Model-Control Group**

**Gross**

The thymus significantly shrank and became jaundice in all rats.

**Pathological Changes under Light Microscopy**

No significant difference in pathological changes was noted among each time point after operation. The thymic tissue was roughly normal in the majority of rats. Obscure boundary between thymic cortex and medulla was occasionally seen. The pathological changes were similar between 21 and 28 days after operation: the thymic tissue was roughly normal in the majority of rats, and obscure boundary between thymic cortex and medulla was occasionally seen.

**(3) Treated Group**

**Gross**

The thymus became slightly jaundice but showed no obvious shrinkage.

**Pathological Changes under Light Microscopy**

No significant difference in pathological changes was noted among each time point after operation. The thymic tissue was normal. Obscure boundary between thymic cortex and medulla was occasionally seen. The thymic tissue was roughly normal in the majority of rats, and obscure boundary between thymic cortex and medulla of was seen in some rats.

#### 3.2.7. Lymph Nodes

**(1) Sham-Operated Group**

**Gross**

The morphology of lymph nodes was normal.

**Pathological Changes under Light Microscopy**

No marked difference in pathological changeswas observed. The morphology and structure of lymph nodes were roughly normal. The enlargement of the follicular germinal centers and the hyperplasia of sinus cells were seen in few rats.

**(2) Model-Control Group**

**Gross**

Lymph nodes became yellow in half of the rats, the texture of lymph nodes showed no changes.

**Pathological Changes under Light Microscopy**

**Figure 12:**
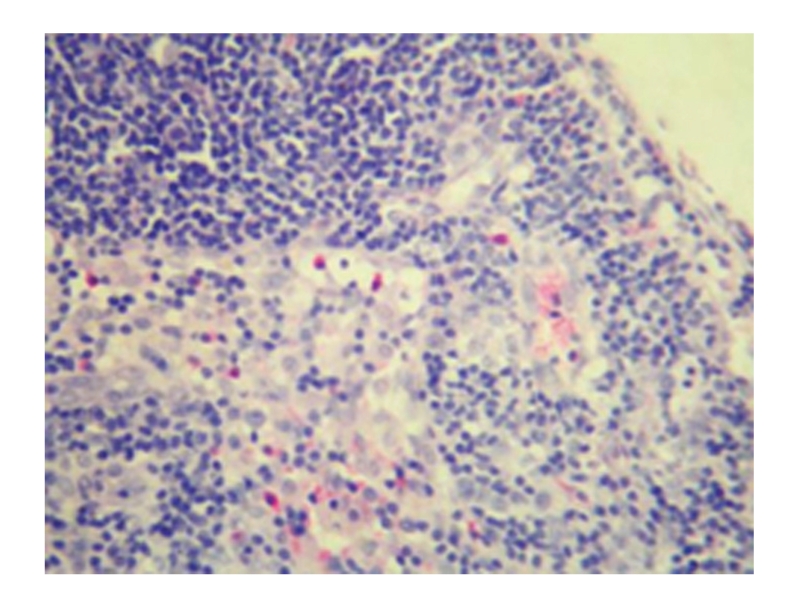
Sham-operated group 28 days; hyperplasia of sinus cell and inflammatory cell infiltration in lymph node; HE × 200.

No marked difference in pathological changes was observed. The enlargement of the follicular germinal centers and the hyperplasia of sinus cells were seen in the majority of rats, and spotty necrosis could be seen in the mantle zone and germinal centers, see Figure 12.

**(3) Treated Group**

**Gross**

The pathological changesshowed no marked difference compared with those in model-control group.

**Pathological Changes under Light Microscopy**

**Figure 13:**
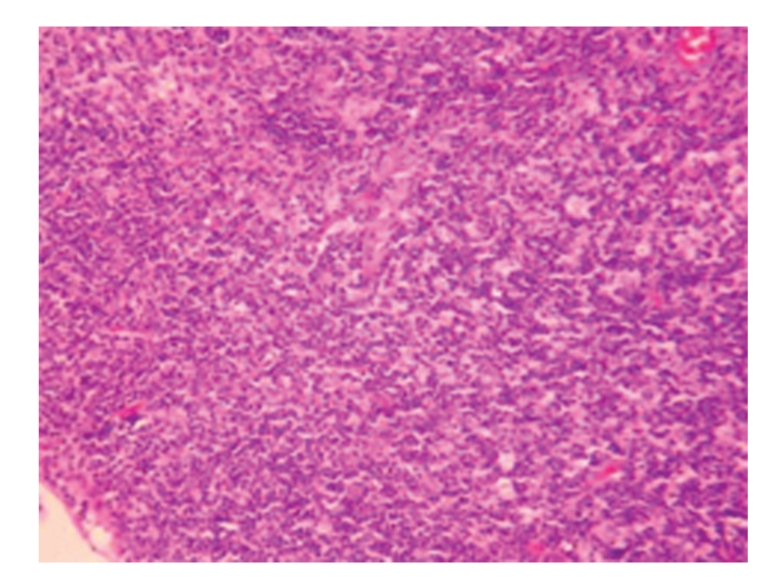
Treated group 28 days; normal lymph node; HE × 100.

No marked difference in pathological changes was observed. The boundary of the follicular germinal centers in lymph nodes was clear. The enlargement of the follicular germinal centers and the hyperplasia of sinus cells were seen in the majority of rats; and few rats showed no obvious pathological changes in lymph nodes, see Figure 13.

### 3.3. Comparison of the Content of Endotoxin in Plasma

On 21st and 28th day, the contents in the sham-operated group were significantly lower than those in model-control group and treated group (*P* < .01), and those of treated group were significantly lower than that in model-control group (*P* < .05), see Table 1.

### 3.4. Comparison of the Content of PLA_2_ in Plasma

On 21 and 28 d, the contents in sham-operated group were significantly lower than those in model-control group and treated group (*P* < .01). On 28th day, the content in treated group was significantly lower than that in model-control group (*P* < .01), see Table 1.

### 3.5. Comparison of the Content of TNF-*α* in Serum

On 21 and 28 d, the contents of serum TNF-*α* in the sham-operated group were significantly lower than those in the model-control group and the treated group (*P* < .01), see Table 1.

## 4. Discussion

When obstructive jaundice (OJ) develops, the hepatic sinusoid is enlarged, endothelial cells swell, and many bacteria and toxins enter into the systemic circulation via hepatic veins and induce endotoxemia. As a result, systemic infection and multiorgan damage were caused [16–18]. In the present study, varying degrees of pathological alterations in multiple organs of obstructive jaundice rats were observed. Under light microscopy, obvious biliary cirrhosis in the liver, congestion and edema in the lung, as well as obvious inflammatory reaction in the intestinal mucosa and kidney were seen. These pathological alterations were identical to those reported in literature. For this reason, it is of important significance to protect these organs in the treatment of OJ.

Some researchers have reported the therapeutic effect of *S. miltiorrhizae* injection against OJ [6, 19]. They found that *S. miltiorrhizae* could significantly improve liver damage in OJ animals, protect intestinal mucosal barrier function in patients with early OJ and promote perioperative recovery of renal function in OJ patients. In the present study, we observed the pathological alterations in multiple organs of OJ rats on days 21 and 28, which represented pathological manifestations of late-sage OJ. *S. miltiorrhizae* showed some protective effects on the majority of organs of rats with late-stage OJ, especially prominent on the liver, kidney and spleen. The protective effects of *S. miltiorrhizae* on the lung, lymph nodes and intestinal mucosa were relatively weak. The observation that *S. miltiorrhizae* had a weak protective effect on the intestinal mucosa may be due to reduced repair capacity for the damaged intestinal mucosa in the late stage of OJ.

During the development process of OJ, an important contributing factor to multiorgan damage is excessive inflammatory reaction mediated by inflammatory mediators such as endotoxin, TNF-*α*, and PLA_2_, OJ is often complicated with intestinal endotoxemia to generate endotoxin that can activate Kupffer cells to produce a large number of inflammatory mediators such as NO, TNF-*α*, and oxygen-free radicals. These inflammatory mediators are involved in inducing functional damage to multiple organs such as the liver and kidney. TNF-*α* is thought to be one of the most critical inflammatory mediators that can mediate endotoxin-induced damage [20–22]. Beside directly acting upon its receptors to induce multiorgan damage, TNF-*α* can also induce the production of IL-1 and IL-6 that can amplify its biological effects to form a cascade reaction and thereby cause the damage to the lung, liver, and intestinal mucosa [16, 18, 23, 24]. An increase in endotoxin and TNF-*α* level can directly alter renal hemodynamics and induce the redistribution of renal blood flow, thereby leading to renal cortical ischemia as well as necrosis of renal tubules and cortex [11, 13, 22]. In animal, OJ can also induce pulmonary edema as well as apoptosis and necrosis of splenic cells, whose extent is positively correlated with the contents of endotoxin and TNF-*α* in the serum [5, 16, 25–27]. PLA_2_ is another important inflammatory mediator contributing to OJ-induced multiorgan damage. It has direct toxic effects on renal tubular epithelial cells and can increase pulmonary capillary permeability [28–30]. Therefore, its activity is correlated with the extent of lung injury [31, 32]. TNF-*α* is able to activate PLA_2_ and, together with endotoxin, is involved in the development and progression of OJ-induced multi-organ damage. At present, it is known that *S. miltiorrhizae* is a traditional Chinese drug that has relatively unambiguous antiendoxin effects and can directly neutralize and destroy endoxin [33–35]. It has been pointed out in some studies that *S. miltiorrhizae* can protect hepatic cells and maintain liver function through reducing inflammatory mediator levels and improving microcirculation [6, 9]. *S. miltiorrhizae* is also able to significantly improve the liver function of OJ patients at the early postoperative stage [36]. In OJ, *S. miltiorrhizae* is able to reduce endotoxemia, regulate the production and secretion of vasoactive substances, and improve renal blood perfusion [37], thereby exerting protective effects against OJ-induced damage to kidney function [10].

In this study, we found that the mortality rates of OJ rats in model-control group were almost two times high as those in treated group, suggesting that *S. miltiorrhizae* injection can indeed reduce the mortality rate of rats. However, no statistically significant difference in the mortality rate of rats was observed, which may be due to the limited sample numbers. We also found that the contents of endotoxin and PLA_2_ of treated group were significantly lower than those in model-control group. However, after careful analysis, we found that the absolute median values of the serum TNF-*α* content of treated group were actually reduced. We guessed that *S. miltiorrhizae* injection can indeed reduce the TNF-*α* content. The main reason for the absence of significant difference may be due to the lower number of experimental rats. In follow-up studies, we plan to increase the number of experimental rats so that the result, that is, consistent with the speculation could be obtained. Thus, we speculate that the protective effects of *S. miltiorrhizae* against OJ-induced multi-organ damage in rats may be associated with reducing the contents of endotoxin, TNF-*α*, and PLA_2_.

Currently, the mechanism underlying the protective effects of *S. miltiorrhizae* against OJ-induced multi-organ damage has not been fully clarified and double-blind control studies of *S. miltiorrhizae* with a large sample size are lacking. Therefore, our future study will be aimed to further explore these relevant issues. This experiment results provided an experimental basis for treatment of OJ patients with *S. miltiorrhizae*.

## Figures and Tables

**Table 1:** Comparison of endotoxin, PLA_2_, and TNF-*α* level.

Indexes	Sham-operated group		Model-control group		Treated group	
	21 d	28 d	21 d	28 d	21 d	28 d
Endotoxin (Eu/mL)	0.2 ± 0.05	0.3 ± 0.04	0.7 ± 0.1**	0.8 ± 0.1**	0.5 ± 0.1** ++	0.5 ± 0.1** ++
PLA_2_ (U/mL)	207.2 ± 38.6	203.0 ± 31.0	701.0 ± 151.7**	738.6 ± 60.2**	593.2 ± 146.3**	645.6 ± 95.1** ++
TNF-*α* (pg/mL)	14.2 ± 3.2	15.6 ± 4.9	31.5 ± 11.0**	57.70 ± 13.0**	24.4 ± 9.2**	44.2 ± 23.1**

Compare to sham-operated group, *P < .05, **P < .01; compare to model-control group, ^+^ *P* < .05, ^++^ *P* < .01.
